# Isolation of an antimicrobial‐resistant, biofilm‐forming, *Klebsiella grimontii* isolate from a reusable water bottle

**DOI:** 10.1002/mbo3.1023

**Published:** 2020-03-03

**Authors:** Alasdair T. M. Hubbard, Enas Newire, João Botelho, Jesús Reiné, Elli Wright, Emma A Murphy, William Hutton, Adam P. Roberts

**Affiliations:** ^1^ Department of Tropical Disease Biology Liverpool School of Tropical Medicine Liverpool UK; ^2^ School of Pharmacy College of Science University of Lincoln Lincoln UK; ^3^ Antibiotic Resistance Evolution Group Max‐Planck‐Institute for Evolutionary Biology Plön Germany; ^4^ Department of Evolutionary Ecology and Genetics Zoological Institute Christian‐Albrechts‐Universität zu Kiel Kiel Germany; ^5^ Department of Clinical Sciences Liverpool School of Tropical Medicine Liverpool UK

**Keywords:** antibiotic resistance, biofilm, environmental, Kleboxymycin, *Klebsiella grimontii*, water bottle

## Abstract

A reusable water bottle was swabbed as part of the citizen science project “Swab and Send,” and a *Klebsiella grimontii* isolate was recovered on chromogenic agar and designated SS141. Whole‐genome sequencing of SS141 showed it has the potential to be a human pathogen as it contains the biosynthetic gene cluster for the potent cytotoxin, kleboxymycin, and genes for other virulence factors. The genome also contains the antibiotic‐resistant genes, *bla*_OXY‐6‐4_, and a variant of *fosA,* which is likely to explain the observed resistance to ampicillin, amoxicillin, and fosfomycin. We have also shown that SS141 forms biofilms on both polystyrene and polypropylene surfaces, providing a reasonable explanation for its ability to colonize a reusable water bottle. With the increasing use of reusable water bottles as an alternative to disposables and a strong forecast for growth in this industry over the next decade, this study highlights the need for cleanliness comparable to other reusable culinary items.

## Graphical abstract

A reusable water bottle was swabbed as part of the citizen science project “Swab and Send,” and a *Klebsiella grimontii* isolate was recovered on chromogenic agar and designated SS141. Whole‐genome sequencing of SS141 showed it has the potential to be a human pathogen as it contains the biosynthetic gene cluster for the potent cytotoxin, kleboxymycin, and genes for other virulence factors and antibiotic resistance. We have also shown that SS141 is a potent biofilm former, providing a reasonable explanation for its ability to colonize a reusable water bottle.

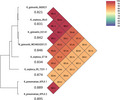

## 1 INTRODUCTION

As the global antimicrobial resistance (AMR) crisis continues and exacerbates, the scientific community are engaged in not only trying to discover novel antimicrobials to treat antimicrobial‐resistant infections (Choi, Hwang, & Lee, 2014; Liu, Yang, et al., 2018) but also understand the drivers of resistance and potential reservoirs of resistant pathogens (Kotsanas et al., 2013; Li et al., 2018; Lowe et al., 2012).

*Enterobacteriaceae* are of increasing concern, so much so that some have been deemed “critical” on the WHO global priority list of antimicrobial‐resistant pathogens. The pathogen *Klebsiella grimontii* is of particular importance as, although it can be part of the normal microflora of the gastrointestinal tract, it has been found to be an etiological agent of antimicrobial‐associated hemorrhagic colitis (Passet & Brisse, 2018). Until recently, *K. grimontii* was thought to be a phylogroup of *Klebsiella oxytoca*; however, it was found to be a distinct pathogenic species of *Klebsiella* and renamed *K. grimontii* and characterized in part by the presence of the chromosomally located β‐lactamase gene, *bla*OXY‐6 (Liu, Feng, et al., 2018; Passet & Brisse, 2018).

Swab and Send (LSTM, 2019) is an antibiotic discovery, citizen science project in which members of the public swab an environment of their choice and return the swab to our laboratory. The bacteria from these swabs are isolated and, using an in‐house assay, the antibiotic‐producing potential of the isolates is determined against indicator strains, including *Micrococcus luteus*, *Escherichia coli*, *Candida auris*, *Candida albicans,* and methicillin‐resistant *Staphylococcus aureus*. As part of the Swab and Send project, a swab was taken from a reusable water bottle from the offices of the Telegraph Newspaper (Ough, 2018). When this swab was plated out on chromogenic agar, there was an abundance of growth of presumptive *Enterobacteriaceae*. Pure culture and sequencing showed it was likely to be an antibiotic‐resistant, pathogenic strain of *K. grimontii*, which is able to form biofilms readily on abiotic surfaces.

## 2 MATERIALS AND METHODS

### 2.1 Media and antimicrobials

Ampicillin (AMP), amoxicillin (AMX), amoxicillin‐clavulanic acid (AMC), ceftriaxone (CEF), olaquindox (OLA), and fosfomycin (FOS) were prepared in molecular grade water; ciprofloxacin (CIP) was prepared in 0.1N hydrochloric acid solution; tetracycline was prepared in 50% ethanol (all Sigma); and chloramphenicol (CHL; Sigma) was prepared in ethanol (VWR) all to a stock concentration of 1 mg/ml.

Growth of SS141 from −80°C stocks was on Lysogeny broth (Lennox) agar at 37°C for 18 hr and subcultured into liquid culture using either Lysogeny broth (Lennox) or cation‐adjusted Mueller Hinton Broth (all Sigma) at 37°C for 18 hr at 200 rpm, unless stated.

### 2.2 Isolation of SS141

A single sample was taken from the interior of the rim of a reusable water bottle using a Transwab^®^ Amies Charcoal Swab (MWE Medical Wire & Equipment) and kept at ambient temperature. The swab was used to directly inoculate CHROMagar™ Orientation (CHROMagar) chromogenic agar plate and incubated at 37°C for 18 hr. A single colony that was metallic green/blue in appearance was chosen and pure‐cultured onto CHROMagar™ Orientation agar. The pure isolate was then kept in stocks at −80°C in 40% glycerol (Sigma, UK).

### 2.3 Sequencing and bioinformatics

Short read sequencing of isolate SS141 and subsequent read trimming were performed by MicrobesNG (MicrobesNG) using the Illumina MiSeq platform. Long read sequencing was performed using the Oxford Nanopore Technologies MinION, using the SQK‐LSK109 ligation and SQK‐RBK103 barcoding kit, on an R9.4.1 flowcell, and all reads were basecalled via MinKNOW. Sequenced reads were demultiplexed, and the adapters were trimmed using Porechop (version 0.2.4) and filtered for a quality score of 30 using Filtlong (version 0.2.0). The whole‐genome sequence of SS141 was assembled using both long and short reads with Unicycler via Galaxy (version 0.4.6.0; Wick, Judd, Gorrie, & Holt, 2017), the quality statistics of each assembly were determined using QUAST (version 4.6.3; Mikheenko, Prjibelski, Saveliev, Antipov, & Gurevich, 2018), and then, subsequently assembled contigs were annotated using Prokka (version 1.14.0; Seemann, 2014). Finally, the assembly graph was visualized using Bandage (version 0.8.1; Wick, Schultz, Zobel, & Holt, 2015).

Using the assembled contigs, it was initially determined whether the isolate was pathogenic using PathogenFinder (version 1.1; Cosentino, Voldby Larsen, Moller Aarestrup, & Lund, 2013) and compared with other strains of *K. grimontii*, *K. oxytoca*, and *Klebsiella pneumoniae* by calculating the average nucleotide identity (ANI) using OrthoANI (version 0.93.1; Lee, Ouk Kim, Park, & Chun, 2016). Acquired antimicrobial resistance genes located in the whole genome sequence or plasmids were searched for using ResFinder (version 3.2; Zankari et al., 2012) with an identity threshold of 60% and a minimum coverage of 60%, and metal resistance genes were identified using the BacMet database (version 2.0; Pal, Bengtsson‐Palme, Rensing, Kristiansson, & Larsson, 2014). Finally, biosynthetic gene clusters (BGC) in the whole genome sequence were searched for using antiSMASH (version 5.0; Blin et al., 2019) and the kleboxymycin biosynthetic gene cluster characterized using SnapGene software (version 3.3.4).

### 2.4 Biofilm assays

The 96‐well plate biofilm assay was performed as described previously (Hubbard, Jafari, Feasey, Rohn, & Roberts, 2019) in a 96‐well plate made from either polystyrene (Costar^®^ round bottom, nontreated, sterile, microplates; Corning Life Sciences) or polypropylene (U‐bottom, sterile 96 well microplate; Greiner Bio‐One). Briefly, following incubation in Luria Bertani broth, each culture was diluted 1/1,000 in M9 (50% (v/v) M9 minimal salts (2×) (Gibco, Thermo Fisher Scientific), 0.4% d‐glucose, 4 mM magnesium sulfate (both Sigma), and 0.05 mM calcium chloride (Millipore) and incubated for 24 hr at either room temperature or 37°C, statically. Following incubation, each culture was washed four times in PBS (PBS, pH 7.2; Gibco, Thermo Fisher Scientific), stained with 0.1% crystal violet (Sigma) followed by washing four times in PBS, and the stained biofilm dissolved in 30% acetic acid (Fisher Scientific). Finally, biofilm production was quantified at an optical density of 550 nm (OD_550_) using a microplate reader.

Single tube biofilm assays were performed in 15‐ml centrifuge tubes made from either polystyrene or polypropylene (Falcon^®^; Corning Life Sciences). Following incubation Luria Bertani broth, each culture was diluted 1/1,000 in 4 ml of M9 and incubated statically at 37°C or room temperature for 24 hr alongside a negative control of 4 ml M9 only. After incubation, each tube was washed four times with 6 ml of PBS and left to dry for 30–60 min and then stained with 5 ml 0.1% crystal violet solution for 15 min. The crystal violet stain was washed off with four washes of 6 ml PBS and left to dry for 60–120 min and photographed.

### 2.5 Minimum inhibitory concentrations

Minimum inhibitory concentration (MIC) of AMP, AMX, AMC, CEF, CIP, OLA, CHL, and FOF to SS141 were carried out using cation‐adjusted Mueller Hinton Broth following the CLSI guidelines for antimicrobial susceptibility testing using the broth microdilution methods (CLSI, 2018).

## 3 RESULTS AND DISCUSSION

The swab taken from a reusable water bottle was initially plated out on to the chromogenic CHROMagar™ Orientation agar routinely used for the identification of urinary tract pathogens (Samra, Heifetz, Talmor, Bain, & Bahar, 1998), which identified a significant amount of presumptive *Enterobacteriaceae* present on the swab. Due to the metallic blue/green pigmentation and morphology of the colonies on the chromogenic agar, it was determined that these colonies were probable *Citrobacter*, *Enterobacter,* or *Klebsiella*, which are often pathogenic. We therefore subcultured until it was a pure isolate, denoted as isolate SS141.

**Figure 1:**
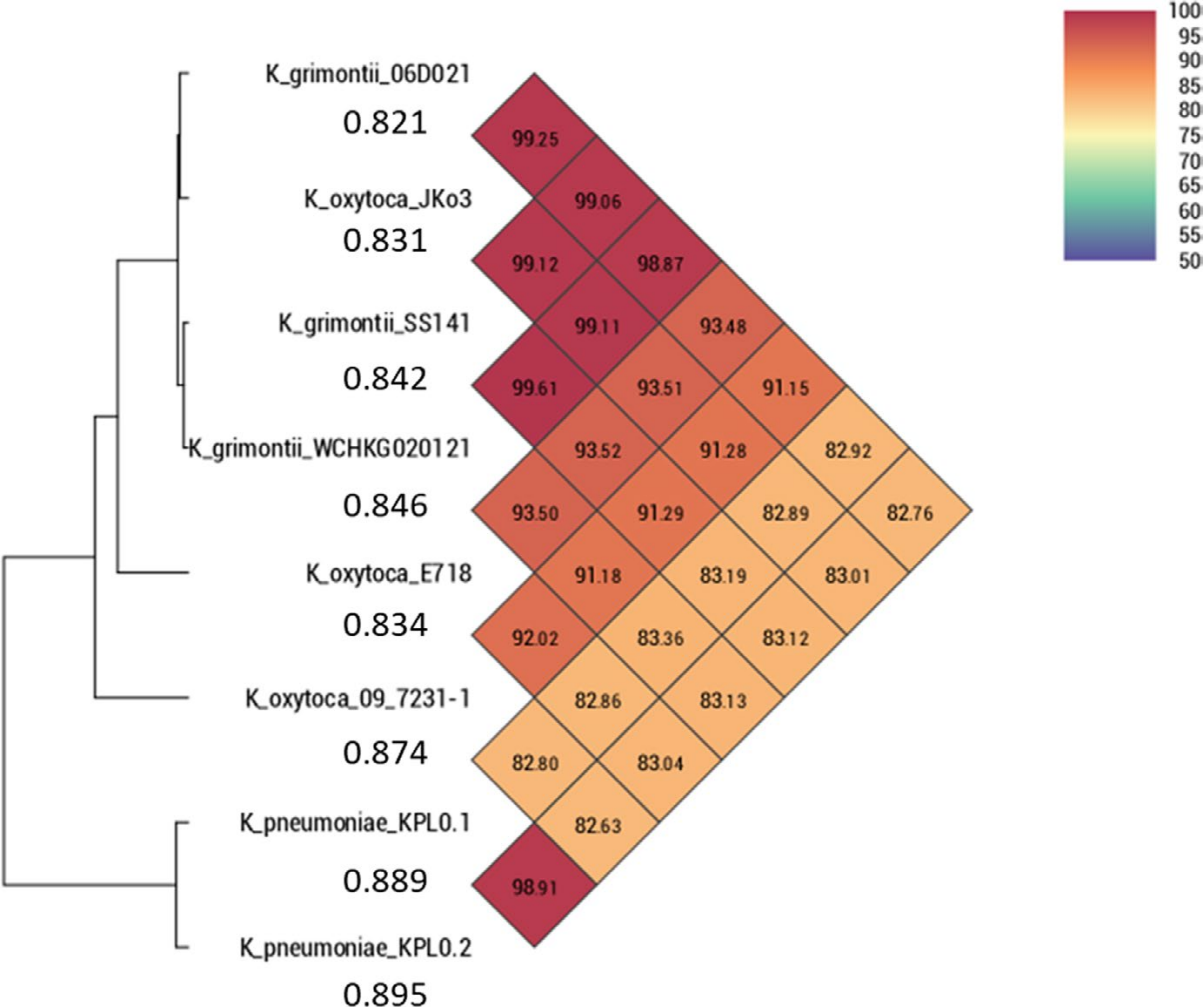
Calculation of the percentage Average Nucleotide Identity (ANI) of *K. grimontii* SS141, *K. grimontii* WCHKG020121, *K. grimontii* 06D021, *K. oxytoca* 09‐7231‐1, *K. oxytoca* E718, *K. oxytoca* JKo3, *K. pneumoniae* KPL0.1, and *K. pneumoniae* KPL0.2. *K. oxytoca E718 was submitted as *Klebsiella michiganensis* on GenBank. Figures under the isolate name represent the probability of being a human pathogen, as determined by PathogenFinder

Following a hybrid assembly of the whole genome sequencing data, the isolate was identified as *K. grimontii.* It contains two low copy number plasmids: IncFII(K) (101 kb in size and 4.11× depth) and IncFIA(HI1) (134 kb in size and 1.98× depth) and a small extrachromosomal molecule, which was unable to be fully resolved and with extremely high copy number (between 1,348.61 and 1,357.92× depth). Antimicrobial resistance genes were not found to be present on any of these plasmids; however, putative heavy metal‐resistant genes (*silE*, *cusA*, *pcoR,* and *copA/B/C/D* potentially conferring resistance to silver and copper) were found to be present on the IncFIA(HI1) plasmid and *arsR*, implicated in resistance to arsenic, was found on the chromosome of SS141. While *K. grimontii* can be asymptomatically carried by humans, it has also been associated with the cause of a number of infections including antibiotic‐associated hemorrhagic colitis and bacteremia (Passet & Brisse, 2018). Using PathogenFinder, we found SS141 is likely to be a human pathogen with a probability score of 0.842. Subsequently, we compared the SS141 genome to a clinical strain isolated from a patient's sputum, *K. grimontii* WCHKG020121 (Liu, Feng, et al., 2018); the reference strain *K. grimontii* 06D021 (Passet & Brisse, 2018); three published genomes of pathogenic strains of *K. oxytoca*: *K. oxytoca* 09‐7231‐1 isolated from a mouse tumor abscess (Darby et al., 2014), *K. oxytoca* E718*, a clinical isolate from Taiwan (Liao et al., 2012), and *K. oxytoca* JKo3, a clinical isolate from a strain collection in Japan (Iwase, Ogura, Hayashi, & Mizunoe, 2016); and two clinical isolates of *K. pneumoniae* (KPL0.1 and KPL0.2; Le Guern et al., 2018) using ANI (Figure 1). We found that SS141 was very closely related to the clinical isolate *K. grimontii* WCHKG020121 (99.61%) and reference strain *K. grimontii* 06D021 (99.06%), further confirming that SS141 is *K. grimontii*. SS141 was also 99.12% identical to *K. oxytoca* JKo3 suggesting that *K. oxytoca* JKo3 is likely to have been misidentified and is actually *K. grimontii* (Figure 1). All these strains had a similar likelihood of pathogenicity as determined by PathogenFinder (Figure 1).

Four antimicrobial resistance genes were identified to be present on the SS141 chromosome using ResFinder, *bla*_OXY‐6‐4_, which confers resistance to β‐lactam antibiotics, including amoxicillin and ticarcillin (Fevre et al., 2005), and is an indicator of *K. grimontii* (Liu, Feng, et al., 2018; Passet & Brisse, 2018). Both *oqxA* and *oqxB,* which are involved in resistance to olaquindox (Hansen, Johannesen, Burmolle, Sorensen, & Sorensen, 2004), an antimicrobial used as a growth promoter in animals (Barber, Braude, Hosking, & Mitchell, 1979), and a variant of the fosfomycin‐resistant gene *fosA* were all also identified and were all previously found to be present on the chromosome of several other *K. grimontii* isolates (Liu, Feng, et al., 2018). Resistance to the β‐lactam antimicrobials AMP and AMX was confirmed (MIC of 16 and 32 µg/ml, respectively); however, SS141 was still sensitive to both AMC (2 µg/ml) and CEF (0.0625 µg/ml) according to EUCAST clinical breakpoints (Table 1). Resistance to fosfomycin was also confirmed with a MIC of 256 µg/ml, which is well over the clinical breakpoint of >32 µg/ml. Despite the identification of *oqxAB* genes in SS141 using ResFinder, the isolate was sensitive to OLA (16 µg/ml) according to the previously determined breakpoint of >64 µg/ml (Hansen et al., 2004; Liu, Wu, et al., 2018; Table 1). While *oqxB2* was confirmed to be present in the genome following annotation using Prokka, we noticed that the *oqxA* gene identified by ResFinder was annotated by Prokka as *bepF* gene, which encodes for the protein BepF, part of the RND‐type efflux pump BepFG, and has been shown to be involved in drug resistance to some degree in *Brucella suis* (Martin et al., 2009). Therefore, lack of confirmation of the presence of *oqxA* in the genome would explain the lack of resistance seen to olaquindox in the phenotypic assays. The *oqxAB* genes have also been reported to be present in the *K. grimontii* WCHKG020121 genome (Liu, Feng, et al., 2018), which align with 100% identity to the *oqxAB* genes identified by ResFinder in SS141. This highlights an important consideration when assessing antibiotic resistance using genomic data and antibiotic‐resistant gene databases as inaccurate annotations may result in antibiotic resistance being incorrectly attributed to an isolate.

**Table 1:** Minimum inhibitory concentrations (µg/ml) of ampicillin (AMP), amoxicillin (AMX), amoxicillin‐clavulanic acid (AMC), ceftriaxone (CEF), ciprofloxacin (CIP), olaquindox (OLA), chloramphenicol (CHL), and fosfomycin (FOF) to the SS141 isolate

	AMP	AMX	AMC	CEF	CIP	OLA	CHL	FOF
SS141	16	32	2	0.03125–0.0625	0.0078125	16	2–4	256

**Figure 2:**
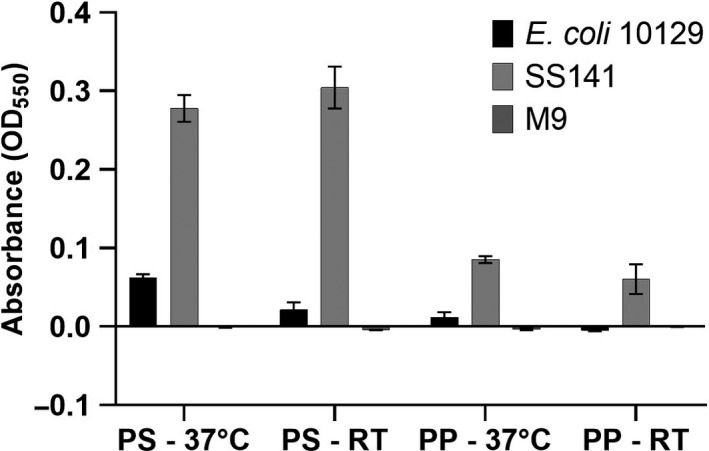
Biofilm formation (expressed as absorbance at OD_550_ of stained biofilm) of the SS141 isolate and *E. coli* 10129 in M9 in polypropylene (PP) and polystyrene (PS) 96 well plates following incubation at either room temperature (RT) or 37°C. Error bars represent the standard error of the mean

**Figure 3:**
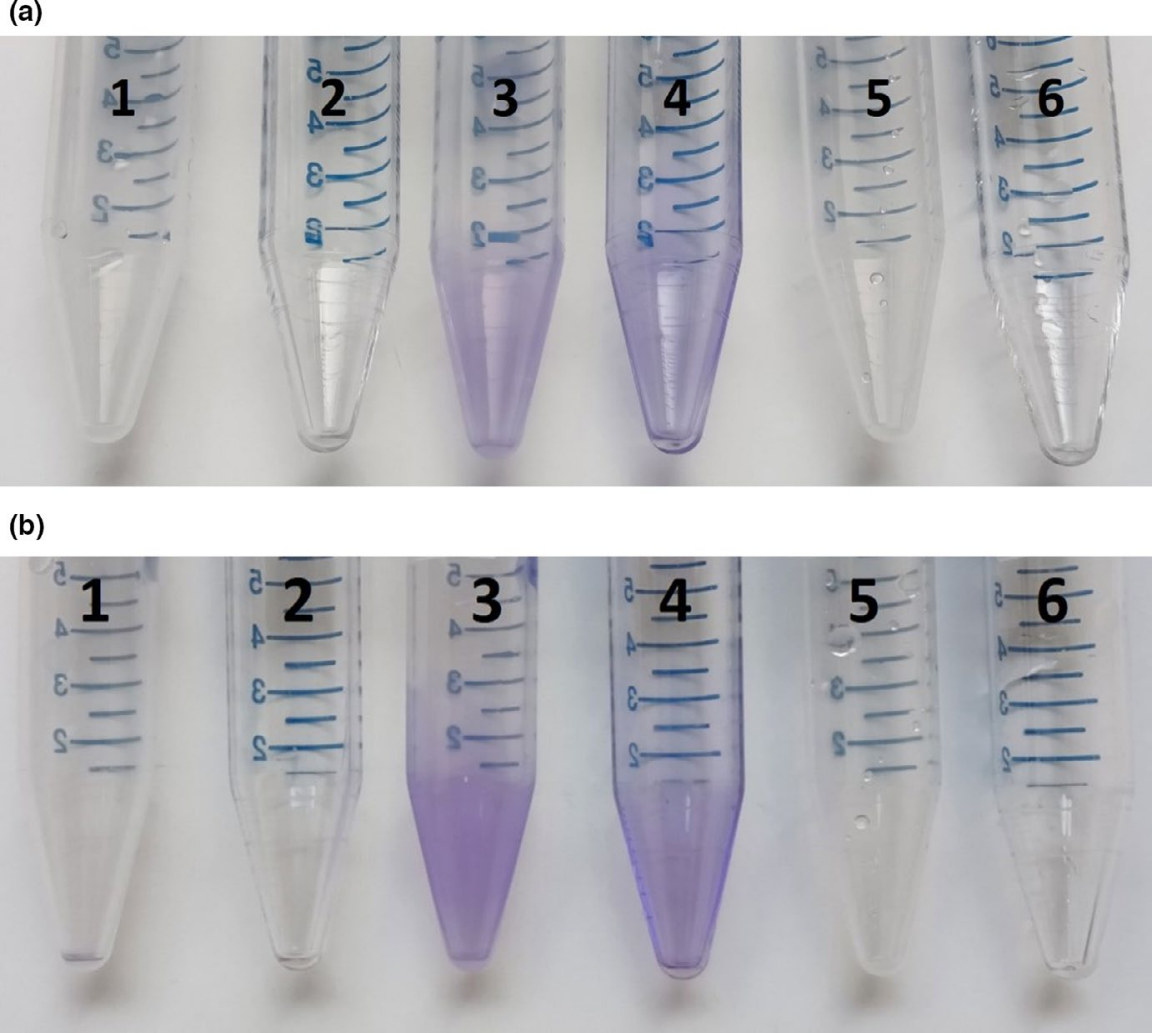
Representative photographs of biofilm formation of (1) *E. coli* 10129 in polypropylene, (2) *E. coli* 10129 in polystyrene, (3) SS141 in polypropylene, (4) SS141 in polystyrene, (5) M9 only in polypropylene, and (6) M9 only in polystyrene incubated at (a) 37°C and (b) room temperature for 24 hr. Repeat 2 and 3 of this experiment are provided in Figures A1 and A2

The SS141 isolate genome contains *treC*, which has previously been shown to be involved biofilm formation in *Klebsiella pneumoniae* and is particularly important during the colonization of the gastrointestinal tract (Wu, Lin, Hsieh, Yang, & Wang, 2011). We have confirmed that SS141 is a biofilm former, producing a visible and significant biofilm during growth in the minimal media M9 at 37°C (Ordinary one‐way ANOVA, uncorrected Fisher's LSD *p*‐value = <.0001) and room temperature (Ordinary one‐way ANOVA, uncorrected Fisher's LSD *p*‐value = <.0001) compared with *Escherichia coli* 10129, a known biofilm producer (Figures 2 and 3a,; Hubbard et al., 2019). As biofilm formation on a polystyrene 96‐well plate and centrifuge tubes do not represent the same plastic commonly used in reuseable water bottles, we also tested the biofilm production potential of SS141 in centrifuge tubes and 96‐well plates made from polypropylene, a significant component of reuseable water bottles. We found that SS141 also produces significant and visible biofilms on polypropylene at both 37°C (Ordinary one‐way ANOVA, uncorrected Fisher's LSD *p*‐value = <.0001) and room temperature (Ordinary one‐way ANOVA, uncorrected Fisher's LSD *p*‐value = .0056; Figures 2 and 3a,) compared with *E. coli* 10129. Although the biofilm production on polypropylene was significantly less than polystyrene at the same temperature (37°C; Ordinary one‐way ANOVA, uncorrected Fisher's LSD *p*‐value = <.0001, room temperature; Ordinary one‐way ANOVA, uncorrected Fisher's LSD *p*‐value = <.0001), this would provide a good explanation for isolation of the *Klebsiella* from the swab and indicates that SS141 has the potential to persist in the environment, in this case on a reusable water bottle. While this study is limited to examining the biofilm production of SS141 on polystyrene and polypropylene surfaces, the ability of bacteria isolated from the environment to form biofilms on a range of plastic surfaces could be investigated, as well as the minimum inoculum size required to form biofilms on plastic surfaces. Finally, the BGC for the potent cytotoxin kleboxymycin was identified using antiSMASH and found to be present within the SS141 genome with 99.53% homology with the previously characterized kleboxymycin BGC (accession number MF401554; Tse et al., 2017). This cytotoxin has been previously associated with the cause of antibiotic‐associated hemorrhagic colitis (Tse et al., 2017), further suggesting that SS141 is a pathogenic strain of *K. grimontii*.

## 4 CONCLUSIONS

We describe the first instance of isolation of a *K. grimontii* isolate from a reusable water bottle. The isolate was found to form biofilms on polypropylene and polystyrene and carried multiple resistance genes to both metals and antibiotics. It is also likely to be pathogenic as the biosynthetic gene cluster for kleboxymycin is present within the genome. There is a strong forecast in the global market for reusable water bottles over the next decade (Accuray‐Research‐LLP, 2019), and this study highlights the aspect of cleanliness when it comes to repeated use of such household items. There is, as yet, no comprehensive scientific study of bacterial colonization of reusable water bottles, and perhaps this is needed to persuade manufacturers and users to promote and practice suitable washing regimens aimed at keeping bacterial load to a minimum.

## CONFLICT OF INTEREST

None declared.

## AUTHOR CONTRIBUTION

Alasdair Hubbard: Conceptualization; Formal analysis; Investigation; Writing‐original draft; Writing‐review & editing. Enas Newire: Formal analysis; Investigation; Writing‐review & editing. João Botelho: Formal analysis; Investigation; Writing‐review & editing. Jesús Reiné: Formal analysis; Investigation; Writing‐review & editing. Elli Wright: Formal analysis; Investigation; Writing‐review & editing. Emma A. Murphy: Formal analysis; Investigation; Writing‐review & editing. William Hutton: Formal analysis; Investigation; Writing‐review & editing. Adam P. Roberts: Conceptualization; Formal analysis; Writing‐original draft; Writing‐review & editing. 

## ETHICS STATEMENT

None required.

## Figures and Tables

**Figure A1:**
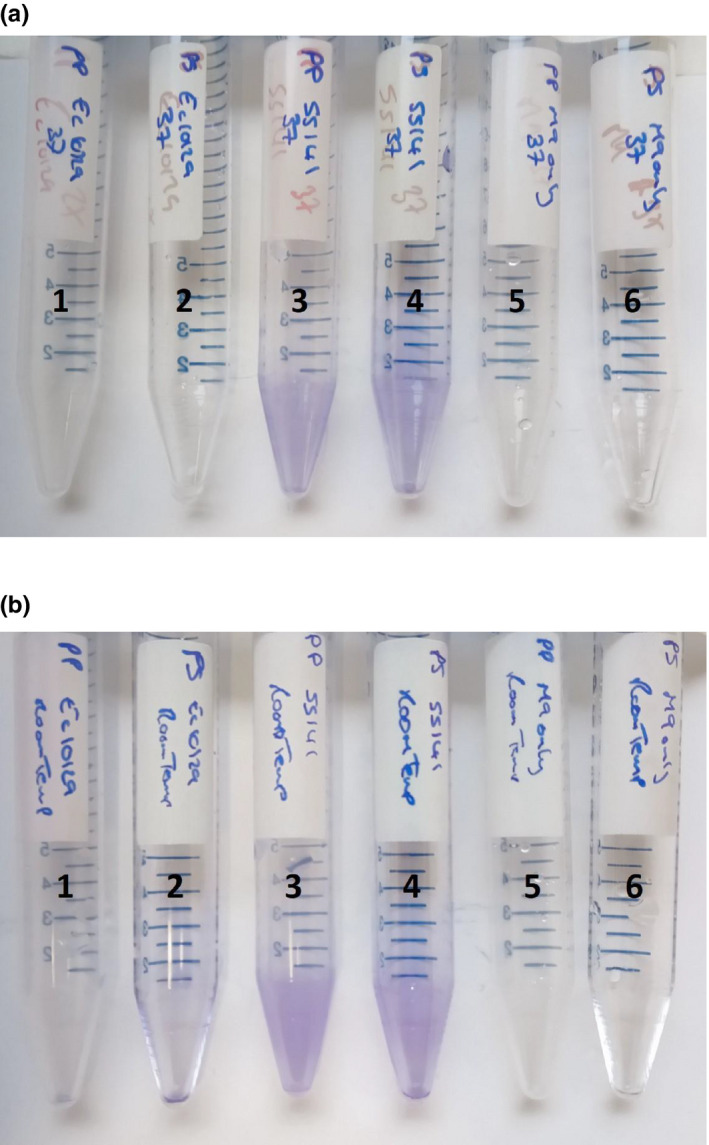
Photographs of the second biological replicate of the 15‐ml tube–based biofilm formation experiment. (1) *E. coli* 10129 in polypropylene, (2) *E. coli* 10129 in polystyrene, (3) SS141 in polypropylene, (4) SS141 in polystyrene, (5) M9 only in polypropylene, and (6) M9 only in polystyrene incubated at (a) 37°C and (b) room temperature for 24 hr

**Figure A2:**
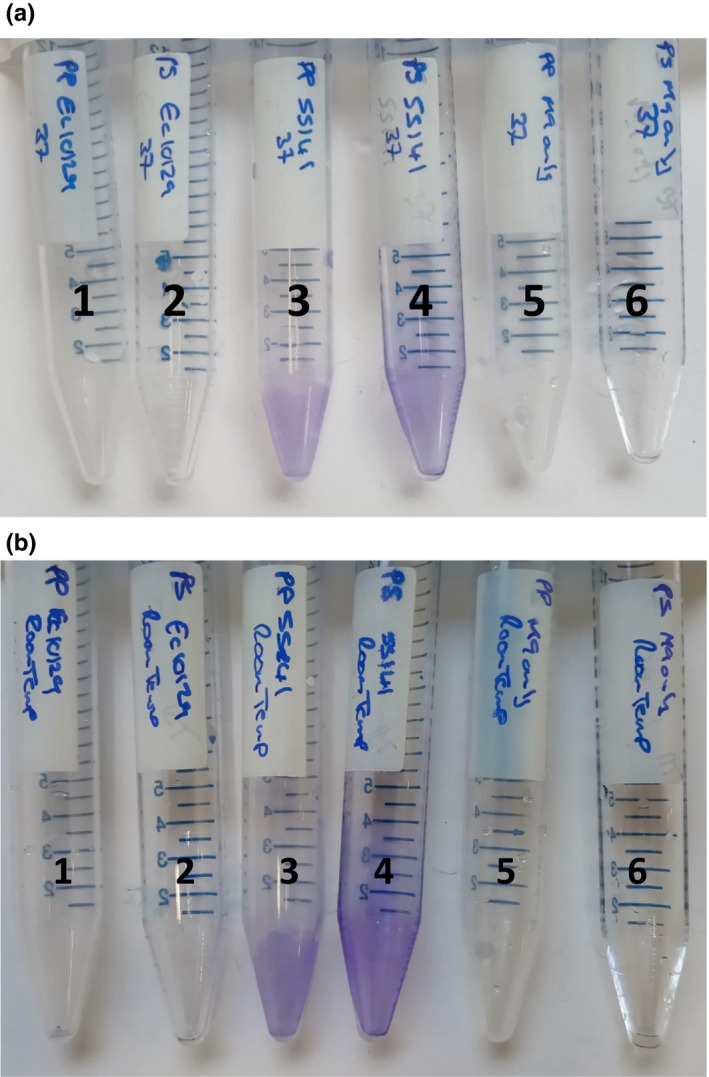
Photographs of the third biological replicate of the 15‐ml tube–based biofilm formation experiment. (1) *E. coli* 10129 in polypropylene, (2) *E. coli* 10129 in polystyrene, (3) SS141 in polypropylene, (4) SS141 in polystyrene, (5) M9 only in polypropylene, and (6) M9 only in polystyrene incubated at (a) 37°C and (b) room temperature for 24 hr

## Data Availability

This Whole Genome Shotgun project for *Klebsiella grimontii* SS141 has been deposited at GenBank under the following accession numbers; complete: circular chromosome accession number CP044527; complete, circular IncFII(K) plasmid accession number CP044529; complete, circular IncFIA(HI1) plasmid accession number CP044528; the incomplete high copy number plasmid accession numbers CP044530 and CP044531.
